# Minimally invasive autopsy as a useful tool for RNA extraction in molecular pathology of lung diseases

**DOI:** 10.1590/1414-431X2025e14745

**Published:** 2025-10-17

**Authors:** S.S. Batah, A.T. Fabro

**Affiliations:** 1 Departamento de Biologia Celular e Molecular e Bioagentes Patogênicos, Faculdade de Medicina de Ribeirão Preto, Universidade de São Paulo, Ribeirão Preto, SP, Brasil

**Keywords:** Minimally invasive autopsy, Molecular pathology, RNA

## Abstract

Minimally invasive autopsy (MIA) is used to understand the pathophysiological mechanisms and clinical outcome of diseases. One of its many advantages is the lower risk of contamination in case of infectious diseases and the quick tissue collection procedure compared to conventional autopsies, which reduces cold ischemia time. Here we investigated the potential role of MIA for molecular pathology studies by comparing RNA yield and quality after RNA extraction from frozen lung tissue samples collected from different methods: MIA, lobectomy, and transbronchial biopsy (TBB). Our results revealed that RNA yield was significantly higher (P<0.05) in the TBB group compared to the lobectomy and MIA groups. However, 93% of MIA samples were non-degraded, showing similar results to TBB, where all had a DV200≥70%. Therefore, MIA proves to be a novel tool for molecular pulmonary pathology in diagnostic and/or research settings.

## Introduction

Autopsy is a medical procedure for: 1) hospital care: to determine the cause of death and investigate other associated diseases (1); 2) teaching and medical training (1); and 3) research: to obtain samples from all tissues for scientific studies to further elucidate disease mechanisms and/or for insights on pathogenesis/treatment. Autopsy was extremely important during the COVID-19 pandemic for understanding pathophysiological mechanisms and clinical outcome.

However, given the biological risk of infectious diseases in conventional autopsies, like COVID-19, and the associated social, cultural, religious, and budgetary issues or even the non-authorization of the invasive procedure by family, minimally invasive autopsy (MIA) emerges as a promising tool (2). For these reasons, we implemented MIA in our hospital during the COVID-19 pandemic.

MIA is a needle-based postmortem examination guided by image methods, such as computed tomography or ultrasound. Although a small amount of tissue is collected, MIA has high accuracy similar to conventional autopsies, as described by Blokker et al. (3), with a higher yield of diagnoses compared to conventional autopsies.

Additionally, an advantage of MIA is the rapid tissue collection compared with conventional autopsies, which reduces the cold ischemia time and allows for immediate freezing for further use in molecular studies, such as DNA/RNA extraction for qPCR and next generation sequencing. For this reason, our hypothesis is that the lung RNA from MIA has sufficient quality to be used in research and diagnosis. Therefore, our aim was to compare the quality of lung RNA using MIA and other types of lung samples.

## Material and Methods

### Sample collection

#### Minimally invasive autopsy (MIA) frozen tissue

Modified MIA was performed in 32 patients at bedside up to 1 h after death by a 3-cm incision on the anterior side of the chest between the fourth and fifth ribs. A matching 14-gauge cutting needle (Magnum, Bard, USA) and a biopsy gun (Magnum, Bard) were used. The samples were then placed in a pre-cooled tube and stored immediately in a -80°C freezer.

#### Non-neoplastic areas of lobectomy (LOBE) frozen tissue

LOBE samples were collected in 7 patients within 30 min and stored immediately in frozen liquid nitrogen.

#### Transbronchial biopsy (TBB) frozen tissue

Post COVID-19 TBB were collected in 8 patients during the biopsy procedure with a matching 14-gauge cutting needle (Magnum, Bard) and a biopsy gun (Magnum, Bard). The samples were immediately stored in frozen liquid nitrogen.

### RNA extraction from frozen samples

RNA extraction from frozen samples was performed using the PureLink RNA Mini Kit (Thermo Fisher Scientific, USA). For LOBE and MIA samples, 60 mg of lung tissue was weighed and homogenized in 1.2 mL of lysis buffer (0.6 mL per 30 mg of tissue) using the Omni TH (Tissue Homogenizer, Omni International, USA) for 30-40 s. For TBB samples, tissue weight ranged from 1 to 10 mg, and homogenization was performed in 0.3 mL of lysis buffer using the same rotor-stator for 30-40 s.

All samples were then centrifuged at 2600 *g* for 5 min at room temperature (RT), and the supernatant was transferred to a new RNase-free tube. One volume of 70% ethanol was added to the homogenate and mixed by vortexing. A 700 µL aliquot of the sample was transferred to a Spin Cartridge (Thermo Fisher Scientific, USA), centrifuged at 12,000 *g* for 15 s at RT, and the flow-through was discarded. The Spin Cartridge was reinserted into the same collection tube, and this step was repeated until the entire sample was processed.

Next, 700 µL of Wash Buffer I was added, followed by centrifugation at 12,000 *g* for 15 s at RT. The flow-through and collection tube were discarded, and the Spin Cartridge was transferred to a new collection tube. Subsequently, 500 µL of Wash Buffer II was added and centrifuged at 12,000 *g* for 15 s at RT, with the flow-through discarded. This step was repeated once more. To ensure membrane dryness, the Spin Cartridge was centrifuged at 12,000 *g* for 1 min at RT before being transferred to a recovery tube.

Elution was performed by adding 100 µL of RNase-free water to the center of the Spin Cartridge, incubating for 1 minute, and centrifuging at 12,000 *g* for 2 min at RT. Finally, the purified RNA was immediately stored at -80°C.

### RNA yield and quality analysis

The following steps were performed for all samples. RNA yield was measured using the Qubit RNA HS Assay Kit (5-100 ng) (Thermo Fisher Scientific) with 1 µL of the extracted RNA sample. RNA quality was then assessed using the Agilent RNA 6000 Pico Kit (Agilent, USA), including DV200 analysis, following the manufacturer's protocol. DV200 analysis indicates the extent of RNA degradation, that is, the proportion of RNA molecules that are 200 nucleotides or more.

**Figure 1:**
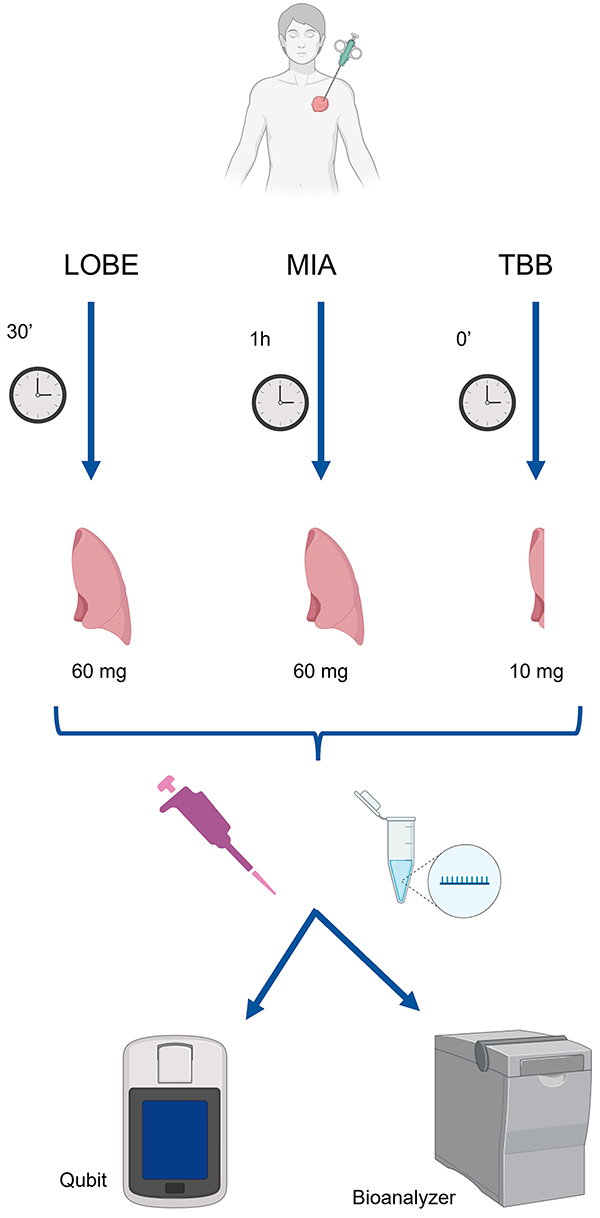
Study design. Different frozen lung tissue collection methods were used, including minimally invasive autopsy (MIA), lobectomy dissection (LOBE), and transbronchial biopsy (TBB). MIA and LOBE samples (60 mg) were collected and frozen 1 h and 30 min post-mortem, respectively, while TBB samples (1-7 mg) were collected and frozen immediately (0'). RNA was extracted using the Purelink RNA Mini Kit (ThermoFisher Scientific) for all samples. RNA yield and quality assessment were performed using Qubit and BioAnalyzer instruments.

Next, to compare the RNA yield among samples collected using different methods, RNA normalization was performed based on the weight of the tissue sample, which varied depending on the collection method. Accordingly, the Qubit result in ng/μL was divided by the sample weight in mg: 60 mg for LOBE and MIA, and 10 mg for TBB. Figure 1 shows the study design.

### Statistical analysis

Statistical analysis was performed with SPSS v.13.0 0 software (SPSS, Inc., IBM, USA). Data were evaluated using unpaired *t*-test. P values less than 0.05 were considered statistically significant.

## Results

**Figure 2:**
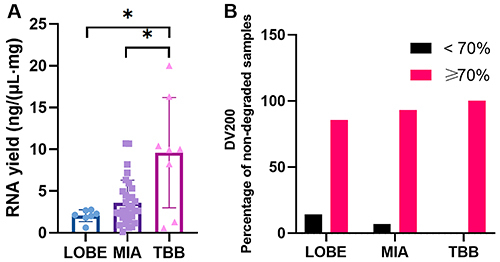
RNA yield and quality from lung samples collected by different methods. **A**, RNA yield was significantly higher for transbronchial biopsy (TBB) compared with lobectomy (LOBE) and minimally invasive autopsy (MIA) (P<0.05). **B**, The majority of the samples were non-degraded (DV200≥70%), meaning that the RNA was intact and equal to or greater than 200 nucleotides. Specifically, 86% of LOBE, 93% of MIA, and 100% of TBB samples met this criterion. Data are reported as means and SD. *P<0.05 (ANOVA).

The evaluation of RNA yield revealed that MIA samples had a higher RNA yield than LOBE. However, frozen biopsy (TBB) samples exhibited a significantly higher (P<0.05) RNA yield compared to both MIA and LOBE (Figure 2A). Additionally, RNA quality assessment using the DV200 metric showed that the majority of samples were intact, with no significant degradation below 200 nucleotides. In fact, 86% of LOBE, 93% of MIA, and 100% of TBB samples were non-degraded, all showing a DV200≥70% (Figure 2B).

## Discussion

We evaluated for first time the yield and quality of RNA from lung samples obtained by three different collection methods. From our results, the importance of cold ischemia time in molecular analysis is highlighted. Each procedure for lung sample collection has a different cold ischemia time, which results in RNAs of different quality, as demonstrated in the literature (4,5). TBB provided the best RNA yield and quality, likely due to the small size of the sample of approximately 10 mg and the immediate and rapid freezing process. In contrast, LOBE samples were larger, around 60 mg, and were not frozen immediately in the operating room. Since these samples were obtained from lung cancer lobectomies, the non-neoplastic and neoplastic tissues had to be separated before freezing.

Surprisingly, MIA samples revealed satisfactory results similar to TBB samples. MIA samples were obtained up to 1 h post-mortem, which could reduce the chances of nuclease contamination, improving DNA/RNA quality. Similarly, Partemi et al. (6) demonstrated that the post-mortem intervals reflect in RNA quality, with samples collected 0.66-2.7 h after death having higher RNA quality than samples collected 36-120 h after death. In addition, other studies that used MIA samples obtained satisfactory and reliable molecular results, including samples collected by MIA from our research group during the COVID-19 pandemic (7-13).

In conclusion, the quantity and quality of RNA from lung samples depend on the type of tissue collection, storage procedure, and time until freezing. Our analysis revealed a surprising result of MIA RNA quantity and quality in many of our publications (7-14). Therefore, MIA could be a novel and useful tool for molecular pulmonary pathology in diagnostics and/or research.

## References

[1] Burton JL, Underwood J (2007). Clinical, educational, and epidemiological value of autopsy. Lancet.

[2] Gaensbacher S, Waldhoer T, Berzlanovich A (2012). The slow death of autopsies: a retrospective analysis of the autopsy prevalence rate in Austria from 1990 to 2009. Eur J Epidemiol.

[3] Blokker BM, Weustink AC, Wagensveld IM, von der Thüsen JH, Pezzato A, Dammers R (2018). Conventional autopsy *versus* minimally invasive autopsy with postmortem MRI, CT, and CT-guided biopsy: comparison of diagnostic performance. Radiology.

[4] Viana CR, Scapulatempo C, Kerr LM, Palmero EI, Marques MMC, Colaiacovo T (2013). The interference of cold ischemia time in the quality of total RNA from frozen tumor samples. Cell Tissue Bank.

[5] Huang J, Qi R, Quackenbush J, Dauway E, Lazaridis E, Yeatman T (2001). Effects of ischemia on gene expression. J Surg Res.

[6] Partemi S, Berne PM, Batlle M, Berruezo A, Mont L, Riuro H (2010). Analysis of mRNA from human heart tissue and putative applications in forensic molecular pathology. Forensic Sci Int.

[7] Batah SS, Rodriguez-Herrera AJ, do Marco MJF, Chiappetto JRS, Gatto M, do Vale SA (2024). Transcriptomic profiling reveals the dynamics of fibrotic progression-related gene expression into post-coronavirus disease 2019 pulmonary fibrosis. Clin Transl Med.

[B08] Batah SS, Zimermam H, Marco MJ, Almeida JP, Wada D, Schnepper A (2023). Whole transcriptome profile underlies the COVID-19 bimodal phenotypes. Eur Resp J.

[B09] Veras FP, Pontelli MC, Silva CM, Toller-Kawahisa JE, de Lima M, Nascimento DC (2020). SARS-CoV-2-triggered neutrophil extracellular traps mediate COVID-19 pathology. J Exp Med.

[B10] Rodrigues TS, de Sa KSG, Ishimoto AY, Becerra A, Oliveira S, Almeida L (2021). Inflammasomes are activated in response to SARS-CoV-2 infection and are associated with COVID-19 severity in patients. J Exp Med.

[B11] Crunfli F, Carregari VC, Veras FP, Silva LS, Nogueira MH, Antunes A (2022). Morphological, cellular, and molecular basis of brain infection in COVID-19 patients. Proc Natl Acad Sci USA.

[B12] Silva CMS, Wanderley CWS, Veras FP, Sonego F, Nascimento DC, Goncalves AV (2021). Gasdermin D inhibition prevents multiple organ dysfunction during sepsis by blocking NET formation. Blood.

[B13] Pontelli MC, Castro IA, Martins RB, La Serra L, Veras FP, Nascimento DC (2022). SARS-CoV-2 productively infects primary human immune system cells in vitro and in COVID-19 patients. J Mol Cell Biol.

[14] Batah SS, Benatti MN, Siyuan L, Telini WM, Barboza JO, Menezes MB (2022). COVID-19 bimodal clinical and pathological phenotypes. Clin Transl Med.

